# Transmission Electron Microscopy Analysis of Skin Lesions from Sporotrichosis Epidemic in Rio de Janeiro, Brazil

**DOI:** 10.4269/ajtmh.14-0401

**Published:** 2015-02-04

**Authors:** Cassio Porto Ferreira, Ana Cristina Oliveira de Almeida, Suzana Corte-Real

**Affiliations:** Leprosy Laboratory, Oswaldo Cruz Institute, Oswaldo Cruz Foundation—FIOCRUZ, Rio de Janeiro, Brazil; Structural Biology Laboratory and Electron Microscopy Platform, Oswaldo Cruz Institute, Oswaldo Cruz Foundation—FIOCRUZ, Rio de Janeiro, Brazil

## Abstract

Transmission electron microscopy can yield useful information in a range of scientific fields; it is capable of imaging at a significantly higher resolution than light microscopes and has been a very useful tool in the identification of morphological changes of the dermis as well as assessment of changes in the extracellular matrix. Our aim is to characterize by electron microscopy the cellular profile of lesions caused by *Sporothrix schenckii* from the sporotrichosis epidemic in its zoonotic form that occurs in Rio de Janeiro, Brazil.

## Report

**Figure 1.**
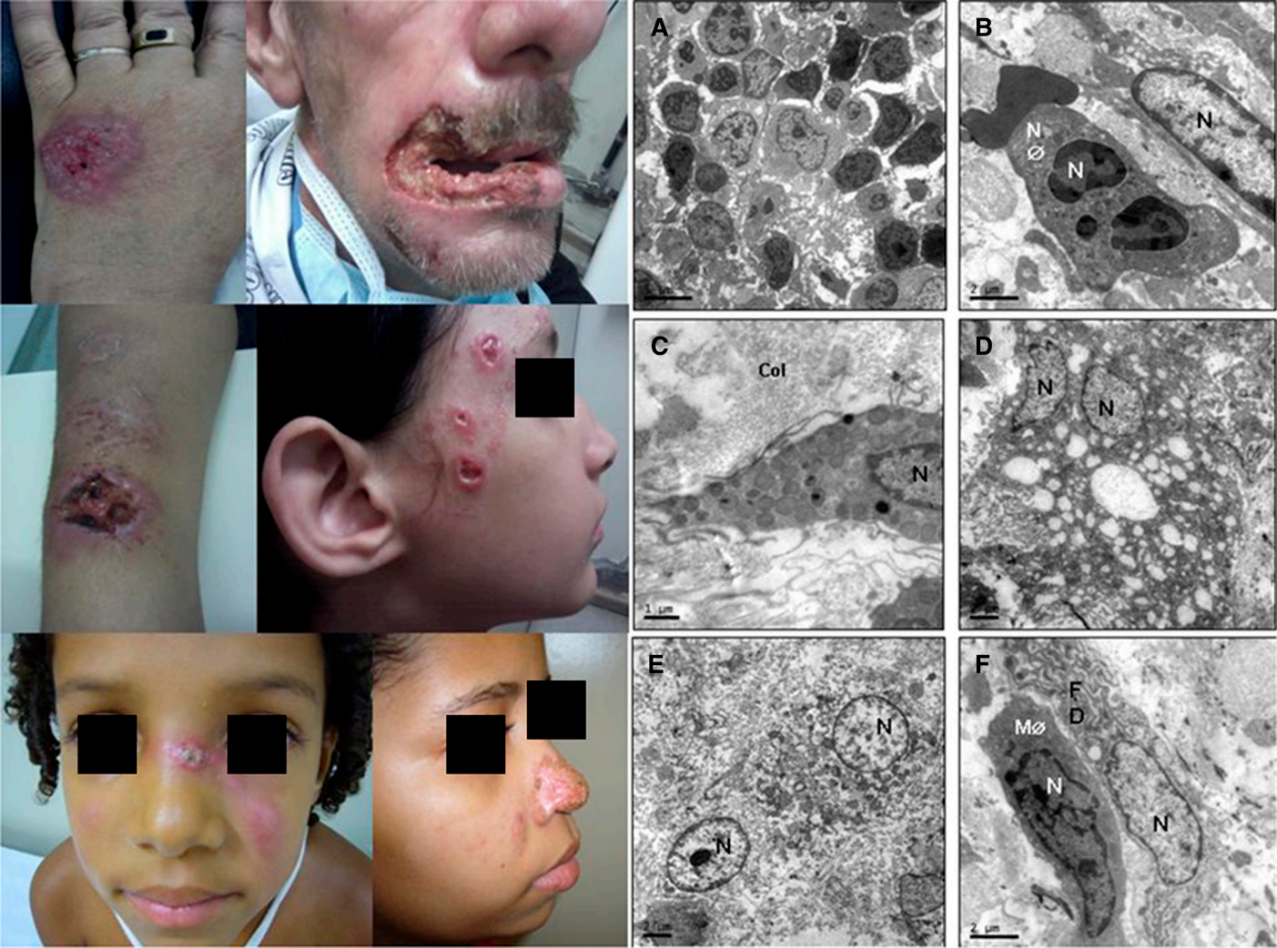
Patients with the sporotrichosis epidemic in Rio de Janeiro, Brazil. Ultrastructural analysis of dermal lesions caused by *Sporothrix schenkii*. (**A** and **B**) Inflammatory infiltrates composed of neutrophils (NØ) and monocytes. (**C**) Degranulated mast cells. (**D**) Large numbers of cells undergoing a process of cell death showing large numbers of vacuoles. (**E**) Cells in necrosis, with loss of cell membrane and organelles disorganization were observed. (**F**) Fibroblasts (FDs) showing well-developed endoplasmic reticulum and mature macrophages (MØs). Col = collagen; N = cell nucleus.

**Figure 2.**
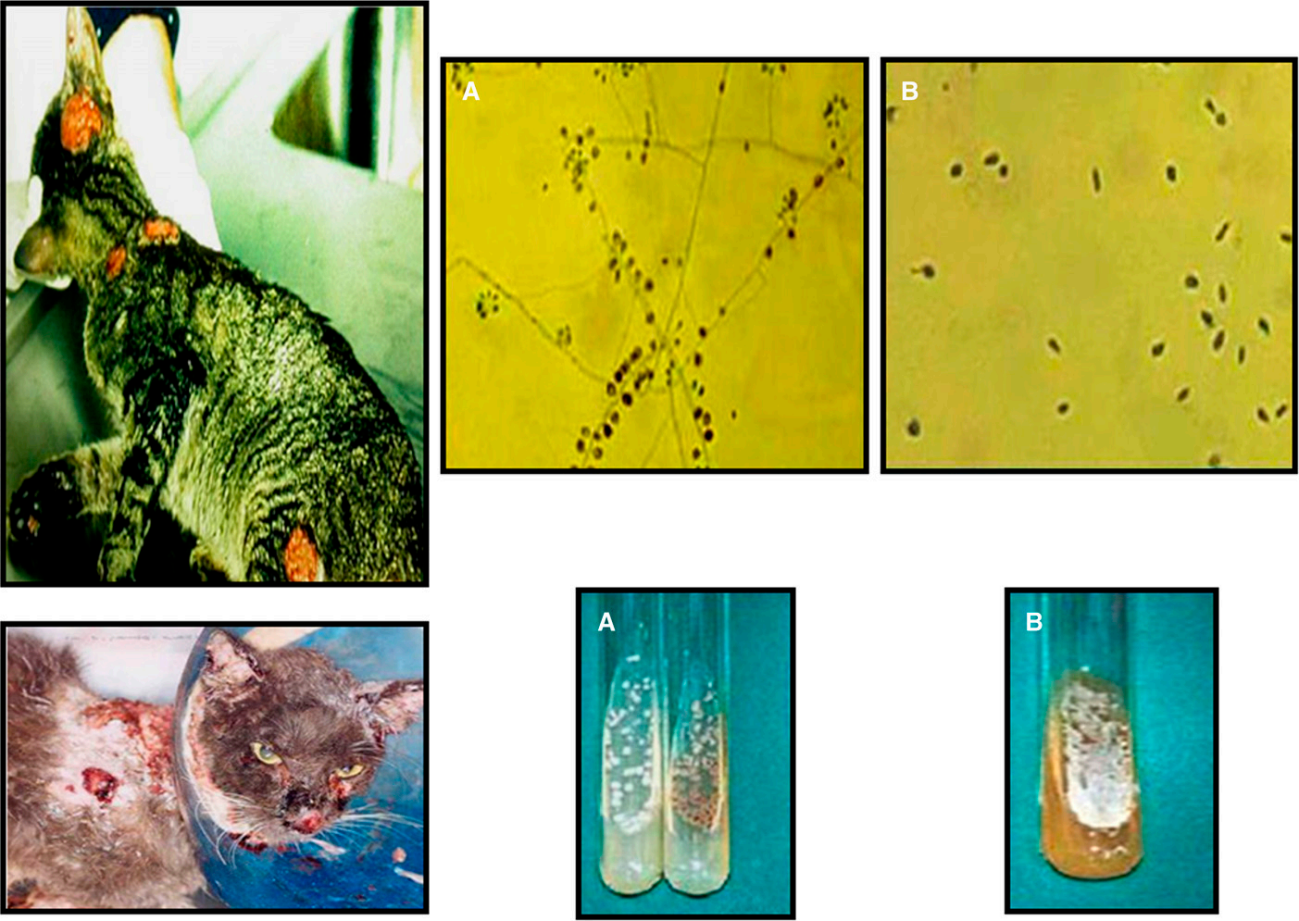
Sick cats with the sporotrichosis epidemic in Rio de Janeiro, Brazil. (**A**) *S. schenckii.* This fungus is dimorphic with a mycelial phase (25°C). (**B**) *S. schenckii.* This fungus is dimorphic with a yeast phase (37°C).

Transmission electron microscopy (TEM) is a microscopy technique in which a beam of electrons is transmitted through an ultrathin specimen and an image is formed from the interaction of the electrons transmitted through the specimen.^1^ Feline transmission of sporotrichosis (Figure 2) was associated with a large and long-lasting outbreak of the disease in Rio de Janeiro, Brazil.^2^ Our goal is to characterize the cellular profile of the injuries caused by *Sporothrix schenckii* through electron microscopy. Thirty skin lesion specimens were collected and prepared as described.^1^ The sections were examined with a Jeol 1011 transmission electron microscope. All patients had isolated *S. schenckii* in culture (Figure 2A and 2B). Our results showed the presence of inflammatory infiltrates composed of monocytes (Figure 1A) and neutrophils (Figure 1B). Mast cells (Figure 1C) and immature and mature macrophages were observed. Lesions were a typical feature, and a large number of cells underwent a process of cell death (Figure 1D), such as necrosis (Figure 1E). It was also observed that a large amount of fibroblasts presented well-developed rough endoplasmic reticulum profiles, thus suggesting an intense production of collagen occupying large areas of the lesion (Figure 1F) that causes fibrosis. Taken together, our data support that TEM is a useful tool that provides both morphological and ultrastructural information as well as matrix changes and cellular components that form the cellular infiltrate in *S. schenckii* infection.
